# Co-cultured methanogen improved the metabolism in the hydrogenosome of anaerobic fungus as revealed by gas chromatography-mass spectrometry analysis

**DOI:** 10.5713/ajas.19.0649

**Published:** 2020-01-13

**Authors:** Yuqi Li, Meizhou Sun, Yuanfei Li, Yanfen Cheng, Weiyun Zhu

**Affiliations:** 1 Laboratory of Gastrointestinal Microbiology, National Center for International Research on Animal Gut Nutrition, Nanjing Agricultural University, Nanjing 210095, China

**Keywords:** Anaerobic Microorganism, Methanogenium, Cytosol, Hydrogenosomes

## Abstract

**Objective:**

The purpose of this study was to reveal the metabolic shift in the fungus co-cultured with the methanogen (*Methanobrevibacter thaueri*).

**Methods:**

Gas chromatography-mass spectrometry was used to investigate the metabolites in anaerobic fungal (*Pecoramyces* sp. F1) cells and the supernatant.

**Results:**

A total of 104 and 102 metabolites were detected in the fungal cells and the supernatant, respectively. The partial least squares-discriminant analysis showed that the metabolite profiles in both the fungal cell and the supernatant were distinctly shifted when co-cultured with methanogen. Statistically, 16 and 30 metabolites were significantly (p<0.05) affected in the fungal cell and the supernatant, respectively by the co-cultured methanogen. Metabolic pathway analysis showed that co-culturing with methanogen reduced the production of lactate from pyruvate in the cytosol and increased metabolism in the hydrogenosomes of the anaerobic fungus. Citrate was accumulated in the cytosol of the fungus co-cultured with the methanogen.

**Conclusion:**

The co-culture of the anaerobic fungus and the methanogen is a good model for studying the microbial interaction between H_2_-producing and H_2_-utilizing microorganisms. However, metabolism in hydrogenosome needs to be further studied to gain better insight in the hydrogen transfer among microorganisms.

## INTRODUCTION

Anaerobic fungi, found in the rumen, hindgut, and feces of ruminants and other herbivores, are important fiber degrading microoganisms [1]. They are assigned to the phylum Neocallimastigomycota and 11 genera have been described so far, namely: *Neocallimastix*, *Caecomyces*, *Piromyces*, *Orpinomyces*, *Anaeromyces*, *Cyllamyces*, *Buwchfawromyces*, *Oontomyces*, *Pecoramyces*, *Feramyces*, and *Liebetanzomyces* [2]. Anaerobic fungi not only produce a variety of fiber degrading enzymes, but also penetrate the plant structure by their rhizoids [3].

Previous studies showed that the fiber degrading ability of anaerobic fungi was significantly improved by co-cultured H_2_-utilizing methanogens [4–6]. This improvement might be due to shift in the metabolic pathways in anaerobic fungi, which increases production of substrates for the co-cultured methanogens and thus reduces production of other metabolites. Bauchop & Mountfort cultivated an anaerobic fungus, *Neocallimastix frontalis*, with H_2_-formate-utilizing methanogen and found that concentrations of lactate, ethanol, and formate were significantly decreased, while the concentration of acetate was significantly increased [5]. During the last decades, similar results have been reported in several studies [6–8].

Using genomic, transcriptomic, and proteomic data, Li et al investigated the shift in the metabolic pathways of anaerobic fungus *Pecoramyces* sp. F1 co-cultured with a syntrophic methanogen [2]. These workers demonstrated that the expression of many genes involved in the metabolic pathway was downregulated by the syntrophic methanogen. However, only a limited number of proteins involved in the metabolic pathway of the anaerobic fungus were downregulated and no significant positive correlation was observed between the transcriptomic data and the proteomic data. The purpose of the study was to investigate the metabolites in an anaerobic fungus (*Pecoramyces* sp. F1) and the metabolic shift that occurs when it is co-cultured with a methanogen (*Methanobrevibacter thaueri*).

## MATERIALS AND METHODS

### Microorganisms and maintenance

The co-culture of anaerobic fungus and methanogen used in the present study was isolated from the rumen of goat [6]. The collection procedure of rumen fluid was approved by the Animals Care and Use Committee of Nanjing Agricultural University (SYXK (SU) 2017–0007). The anaerobic fungus was *Pecoramyces* sp. F1 [2] and the methanogen was *Methanobrevibacter thaueri* [6]. The co-culture was maintained in a medium containing rumen fluid with 1% (w/v) rice straw as a substrate and transferred every 3 days in the fresh medium [8]. The culture medium contained penicillin (1,600 U/mL) and streptomycin (2,000 U/mL) to inhibit growth of bacteria.

The monoculture of the anaerobic fungus was obtained by adding chloramphenicol at a final concentration of 50 mg/L, which inhibits growth of methanogens and bacteria in the co-culture [8]. The monoculture was transferred every 3 days in the same medium as used for the co-culture.

### Experimental design and sample collection

For the experiment, a modified medium M2, without rumen fluid, was used with 20 mM glucose as the substrate [9]. The medium (90 mL) after adjusting pH to 6.8 was anaerobically dispensed into a serum bottle (180 mL capacity). The serum bottle with 100% CO_2_ headspace was sealed with butyl rubber stopper and aluminum crimp to maintain anaerobic condition [10]. Ten bottles were used for mono- and co-cultures, respectively (i.e. a total of 20 bottles), with 5 replicates each for the experimental group and the control group. The experimental group was inoculated with 10 mL of the 3-day old culture and the control group was inoculated with 10 mL of the blank medium. Thereafter, the bottles were incubated at 39°C for 72 h without shaking [8]. After inoculation, the air pressure in the fermentation bottle was balanced with a pressure converter to make the initial air pressure 0. At the end of the fermentation, samples of the supernatant and the fungal cells were collected according to the methods of Marvin-Sikkema et al [11]. The bottles were centrifuged at 200 g for 10 min to separate the fungal cells and the supernatant. The fungal cells were washed using 20 mM phosphate-buffered saline (PBS) containing 20 mM dithiothreitol and centrifugated at 200 g for 10 min. The procedure was repeated twice, and the pellet was dissolved in 2 mL PBS. To break the cells, 1 mm glass beads were added, and the mixture was vortexed for 10 min. It was then centrifuged at 500 g for 15 min. The supernatant was collected to obtain cell contents. The fungal supernatant was centrifuged at 13,000 g for 10 min before the analysis. The samples were stored at −80°C for the gas chromatography-mass spectrometry (GC/MS) analysis.

### Sample preparation and gas chromatography-mass spectrometry analysis

To an aliquot of 50 μL of the cell content or the fungal supernatant was added 200 μL methanol containing 12.5 μg/mL myristic-1,2-^13^C_2_ acid as an internal standard (IS). The mixture was vortexed for 5 min, placed at 4°C for 1 h, and centrifuged at 20,000 g for 10 min at 4°C. An aliquot of 100 μL supernatant was transferred into a GC vial and evaporated to dryness under vacuum (Thermo Fisher Scientific, Asheville, NC, USA). An aliquot of 30 μL methoxyamine in pyridine (10 mg/mL) was added and vortexed for 3 min. After 16 h of methoximation reaction at room temperature, 30 μL of N-methyl-N-(trimethylsilyl) trifluoroacetamide containing 1% trimethylchlorosilane was added, vortexed for 1 min, and placed at room temperature for 1 h for trimethylsilylation. Finally, 30 μL methyl myristate in heptane (30 μg/mL) was added and vortexed, and an aliquot of 0.5 μL was used for the GC/MS analysis.

The analysis of metabolites was conducted by a GC/MS system (SHIMADZU GC/MS QP2010Ultra/SE, Kyoto, Japan) fitted with a RTx-5MS capillary column (30 m×0.25 mm, Agilent J&W Scientific, Folsom, CA, USA). The conditions used for GC/MS analysis were as follows: Helium as carrier gas at flow rate of 1.5 mL/min; initial column temperature of 70°C for 3 min followed by increasing from 70°C to 300°C at a rate of 20°C/min and held for 3 min; transfer line temperature of 205°C and ion source temperature of 200°C; ion source voltage of −70 eV and acceleration voltage of −950 V; and solvent delay of 300 s. The MS data were acquired over the range between m/z 50 – 800 [12].

### Data collection and processing

After rejecting the peaks with signal-to-noise (S/N) lower than 30 and normalizing with IS, the retention time, retention index, and mass spectra of each peak were obtained and compared with the reference standards and database including NIST library (2008), Wiley library, and in-house database established by China Pharmaceutical University [12,13]. The partial least squares-discriminant analysis (PLS-DA) was conducted using the R package *ropls* [14] and significant difference between two groups was considered at p<0.05 and variable importance plot (VIP)>1. Data visualization was done in R studio (www.rstudio.com).

## RESULTS

### Effects of the co-cultured methanogen on metabolic changes in the fungal cell

**Figure 1:**
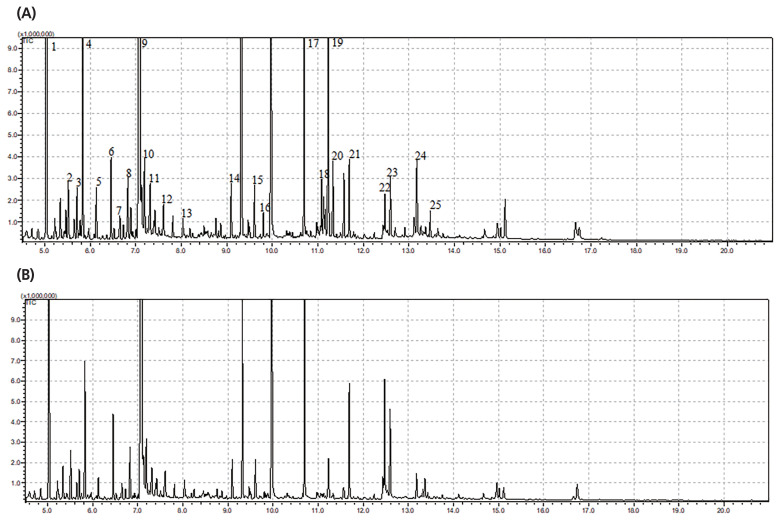
Typical gas chromatography-mass spectrometry (GC/MS) chromatograms of metabolites in the fungal cells with/without the co-cultured methanogen. (A) Monoculture of the anaerobic fungus. (B) Co-culture of the anaerobic fungus and the methanogen. 1, lactate; 2, alanine; 3, Ua3; 4, oxalate; 5, α-aminobutyric acid; 6, 2-keto-3-methylvaleric acid; 7, valine; 8, carbamic acid; 9, leucine; 10, glycerol-3TMS; 11, isoleucine; 12, fumaric acid; 13, threonine; 14, UN01; 15, lauric acid; 16, taurine; 17, internal standard; 18, histidine; 19, glucose; 20, tyrosine; 21, palmitic acid; 22, oleic acid; 23, stearic acid; 24, UI22; 25, UI23.

**Figure 2:**
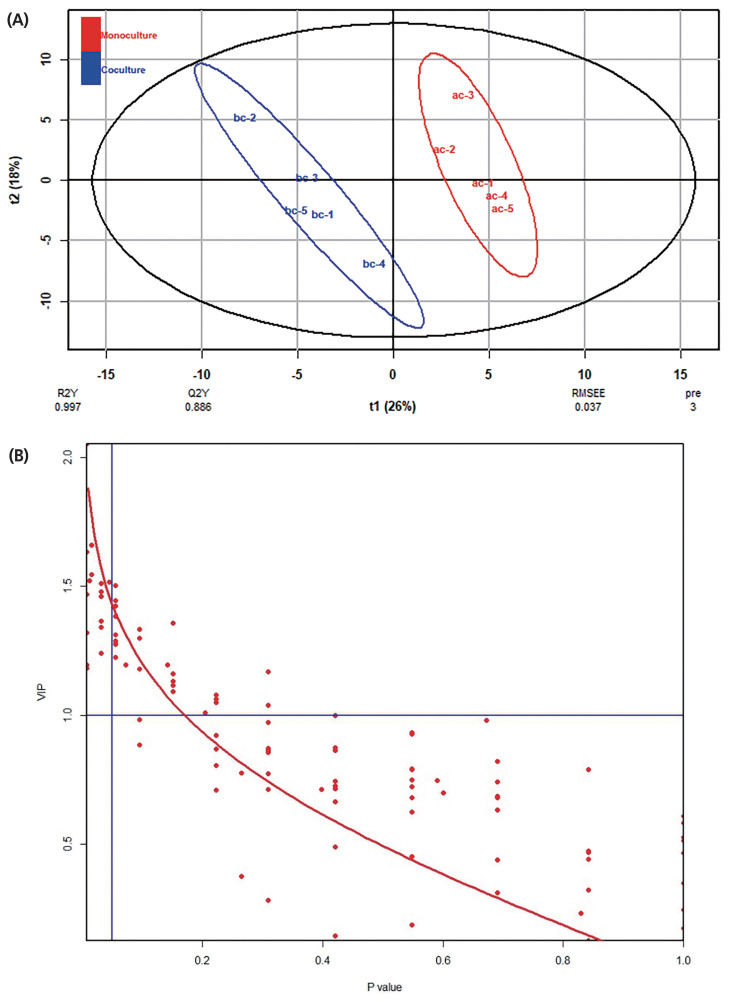
Effects of the co-cultured methanogen on the metabolites in the fungal cell, as evaluated by the PLS-DA analysis. (A) PLS-DA score plot. (B) Relationship between VIP and p-value. PLS-DA, partial least squares-discriminant analysis; VIP, variable importance plot.

The typical GC/MS chromatograms of metabolites in cells of the anaerobic fungus with/without the methanogen are shown in Figure 1. A total of 104 metabolites were detected in the fungal cell and a distinct difference of the metabolite profiles between the two groups was observed in the PLS-DA score plot (Figure 2A). For evaluation of the model, the R2X, R2Y, and Q2 (cum) were calculated as 0.532, 0.997, and 0.886, respectively. Statistical analysis showed that 16 metabolites differed significantly between the two groups (p<0.05) (Figure 2B). As shown in Table 1, 8 metabolites significantly increased in the co-culture of the anaerobic fungus and the methanogen including 6-hydroxy-9H-purine, citric acid, threonine, glutamic acid, isoleucine, monostearin, uracil and valine. The remaining 8 metabolites were significantly decreased, including D-galactono-1,4-lactone, glucose, ribopyranose, beta-D-methylglucopyranoside, ribonic acid and three unknown metabolites. Many of these amino acids participate in cytosol metabolism, and citric acid is an important intermediate in the oxidation pathway of the tricarboxylic acid (TCA) cycle in cytosol metabolism.

### Effects of the co-cultured methanogen on metabolic changes in the fungal supernatant

**Figure 3:**
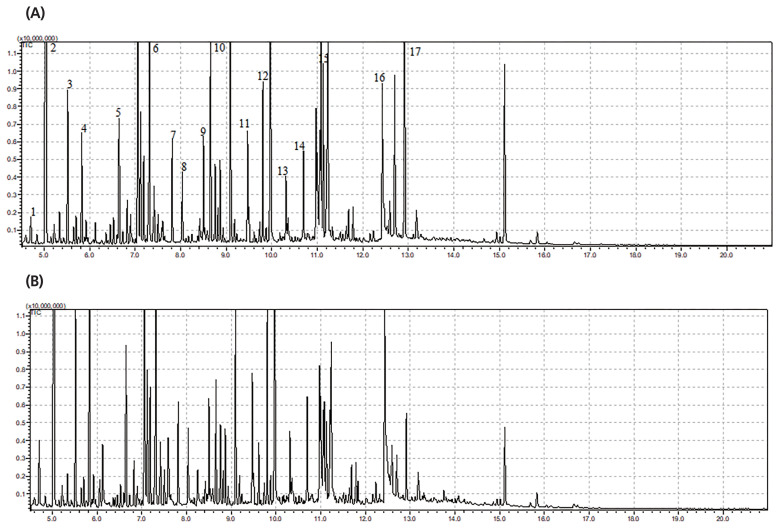
Typical gas chromatography-mass spectrometry chromatograms of metabolites in the supernatant of the anaerobic fungus with/without the co-cultured methanogen. (A) Monoculture of anaerobic fungus. (B) Co-culture of anaerobic fungus and methanogen. 1, pyruvic acid; 2, lactate; 3, alanine; 4, oxalate; 5, urea; 6, isoleucine; 7, serine; 8, threonine; 9, aminomalonic acid; 10, malic acid; 11, glutamic acid; 12, taurine; 13, glycerol-3-phosphate; 14, internal standard; 15, glucose; 16, linoleic acid; 17, cystin.

**Figure 4:**
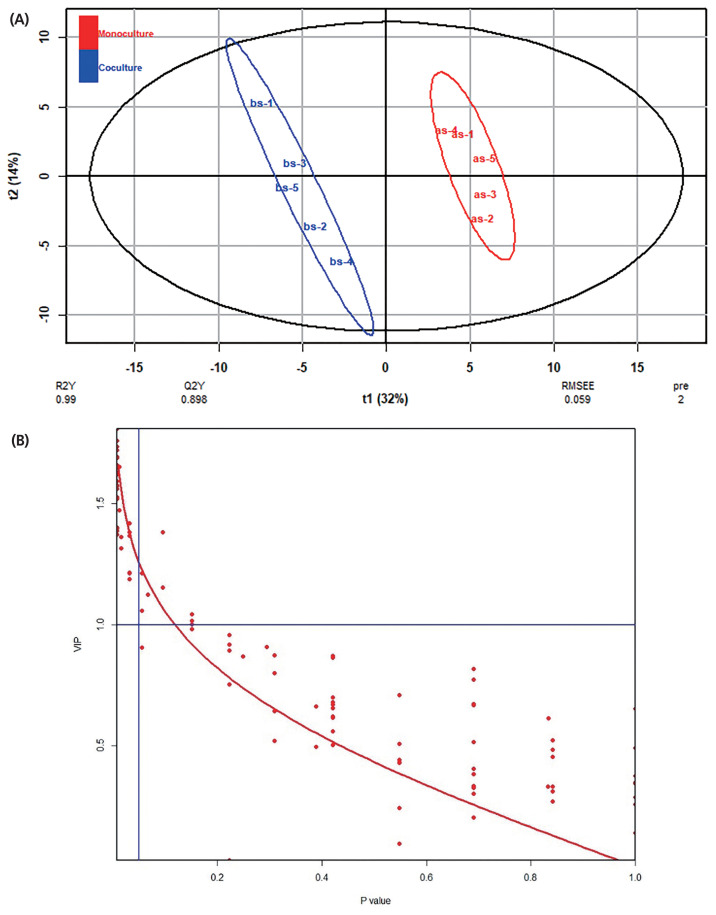
Effects of co-cultured methanogen on the metabolites in the supernatant of cultures as evaluated by PLS-DA analysis. (A) PLS-DA score plot. (B) Relationship between VIP and p-value. PLS-DA, partial least squares-discriminant analysis; VIP, variable importance plot.

The typical GC/MS chromatograms of metabolites in supernatant of the anaerobic fungus with/without the methanogen are shown in Figure 3. A total of 102 metabolites were detected in the supernatant of the cultures and a distinct difference in the metabolite profiles between the two groups was observed in the PLS-DA score plot (Figure 4A). The R2X, R2Y, and Q2 (cum) of the model were 0.453, 0.99, and 0.898, respectively. Statistical analysis showed that 30 metabolites differed significantly between the two groups (Figure 4B). As shown in Table 2, 22 metabolites were significantly increased in the co-culture of the anaerobic fungus and the methanogen and the remaining 8 metabolites were significantly decreased.

### Effect of the co-cultured methanogen and the anaerobic fungus on the metabolic pathway of the fungus

**Figure 5:**
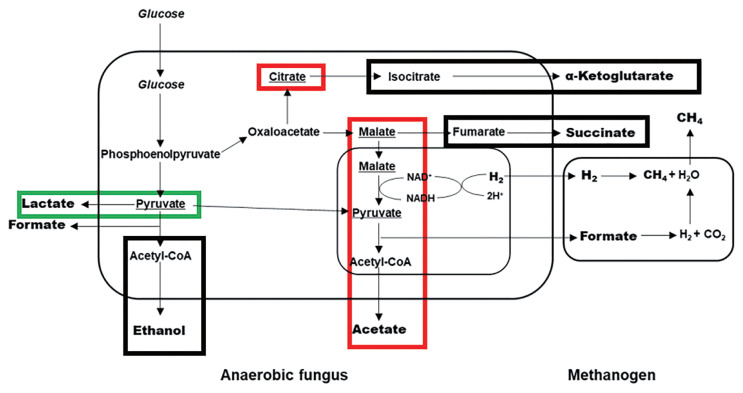
Effects of the co-cultured methanogen on the metabolic pathway of the anaerobic fungus. Metabolic pathway was drawn based on previous reports by Cheng et al [8], Li et al [15], and Li et al [21]. Red, stimulated pathways; green, inhibited pathways; black, unaffected pathways.

Based on the metabolite profiles in the cells of the anaerobic fungus with/without the co-cultured methanogen and previous reports [2,15], the metabolic pathway of the anaerobic fungus is shown in Figure 5. Combined with the previous research conducted in our laboratory, including the study of the metabolic pathway differences in the utilization of glucose by *Pecoramyces* sp. F1 in monoculture and co-culture with the methanogen, as well as the research on the effect of the co-cultured methanogen on the dynamic profile of intermediate and end metabolic pathway of *Pecoramyces* sp. F1, we conclude that co-cultured methanogen stimulated the metabolic pathway to citrate and malate in the cytosol of the anaerobic fungus and stimulated the metabolism in its hydrogenosome.

## DISCUSSION

In the recent past, several studies have focused on the effects of culturing anaerobic fungi with methanogens. For example, several workers [4–7,16] have shown higher fiber degrading ability of anaerobic fungi when co-cultured with methanogens as compared with when they were grown as a monoculture. So far, approximately 60 co-cultures of anaerobic fungi and methanogen combinations have been isolated from different herbivores [6,17–20]. The co-culture of *Pecoramyces* sp. F1 and *Methanobrevibacter thaueri*, used in the present study, is one of such co-cultures, which were isolated from the rumen of Chinese local goat and demonstrated to have higher fiber degrading ability than others [6]. The anaerobic fungus in the co-culture was identified as *Piromyces* sp. F1 according to its morphology and renamed as *Pecoramyces* sp. F1 by using molecular techniques [2].

Present study showed that the metabolic pathways in the anaerobic fungal cell were shifted by the co-cultured methanogen. The pathways in the cytosol were inhibited and the pathways in the hydrogenosome were stimulated, resulting in increased provision of H^+^ for the co-cultured methanogen. The production of lactate from pyruvate in the cytosol decreased in the supernatant by 15%, which is a process of H^+^ consumption (from nicotinamide adenine dinucleotide to nicotinamide adenosine denucleotide) [8,15,21]. The anaerobic fungus does not possess a complete TCA cycle in the cytosol but has reductive (from oxaloacetate to succinate) and oxidative (from citrate to α-ketoglutarate) branches of it [22,23]. Present study showed that citrate was accumulated in the fungal cytosol when co-cultured with the methanogen, which was revealed by the increase of citrate in the fungal cell (7.62-fold). This is consistent with our previous report that citrate was accumulated in the co-culture of the anaerobic fungus and the methanogen [22]. However, no observed (p = 0.265) increase of α-ketoglutarate in the cytosol of the anaerobic fungus co-cultured with the methanogen implies that the oxidative branch was not affected by the co-cultured methanogen, except for citrate accumulation. The content of fumarate in the fungal cells between two groups was not significantly different (p = 1), and even an increase in malate was observed (3.305-fold in the cell, p = 0.056).

Previous studies reported significant accumulation of acetate in the supernatant of the anaerobic fungus co-cultured with the methanogen [6–8], which implied that the metabolism in the hydrogenosomes of the anaerobic fungus was stimulated by the co-cultured methanogen. This was proved in the present study by increase in the contents of malate and pyruvate observed in the fungal cell, also the content of malate in the anaerobic fungal cell was lower than that in the anaerobic fungal cell co-cultured with the methanogen, which indicated increased malate availability in the reductive pathway of the TCA cycle in presence of the methanogen, and enhanced reductive pathway in the cytosol of the anaerobic fungus. A higher amount of malate entered the hydrogenosomes and increased formate and H_2_ production for utilization by the methanogen, thus promoting the metabolism in the hydrogenosomes. Hydrogenosomes are membrane-bound organelles, which have been found in a variety of unicellular anaerobic eukaryotes such as *Trichomonas vaginalis*, *Psalteriomonas lantern*, and *Neocallimastix* [24,25]. Hydrogenosomes are notable for the energy metabolism of anaerobic eukaryotes. They cannot use oxygen but reduce protons to H_2_, which can be used by co-cultured methanogens to produce methane instantly. The stimulation of the metabolism in the hydrogenosome implies higher H_2_ production for the co-cultured methanogen, which benefits both the anaerobic fungus (reduction in growth inhibition from fermentation end products) and the co-cultured methanogen (provision of increased amounts of growth substrate). However, the H_2_ transfer between the anaerobic fungus and the co-cultured methanogen needs to be further studied to obtain better insight into the metabolic interaction among microorganisms.

The co-cultured methanogen reduced the production of lactate from pyruvate in cytosol of the anaerobic fungus and stimulated metabolism in hydrogenosome of the anaerobic fungus. Citrate was accumulated in the fungal cytosol co-cultured with the methanogen. The co-culture of anaerobic fungi and methanogens is a good model for studying the microbial interaction between H_2_-producing and H_2_-utilizing microorganisms. However, the hydrogenosome and its metabolism need to be further studied to evaluate the hydrogen transfer among microorganisms.

## Figures and Tables

**Table 1:** Significantly changed metabolites in the anaerobic fungal cells by the co-cultured methanogen

Compounds	Fold change (bc/ac)	p-value1)	VIP1)
6-Hydroxy-9H-purine	2.432	0.008	1.633
Citric acid	7.620	0.008	2.053
D-Galactono-1,4-lactone	0.189	0.008	1.320
Glucose	0.092	0.008	1.470
Ribopyranose	0.042	0.008	1.182
S07	0.139	0.008	1.195
Beta-D-Methylglucopyranoside	0.094	0.012	1.524
Threonine	1.443	0.016	1.547
UI23	0.298	0.016	1.660
Glutamic acid	1.963	0.032	1.365
Isoleucine	1.680	0.032	1.512
Monostearin	1.497	0.032	1.342
S04	0.654	0.032	1.241
Uracil	1.869	0.032	1.480
Valine	1.870	0.032	1.462
Ribonic acid	0.133	0.045	1.516

bc, fungal cell of the co-culture of anaerobic fungus and methanogen; ac, fungal cell of the anaerobic fungal monoculture; VIP, variable importance plot.

1) When p<0.05 and VIP>1, there is a significant difference in metabolites between bc and ac.

**Table 2:** Significantly changed metabolites in supernatant of the anaerobic fungus by the co-cultured methanogen

Compounds	Fold change (bs/as)	p-value1)	VIP1)
6-Hydroxy-9H-purine	0.575	0.008	1.688
Alanine	1.334	0.008	1.692
Carbamic acid	1.323	0.008	1.396
Cystin	0.105	0.008	1.809
Fructose	0.157	0.008	1.627
Glutamic acid	1.235	0.008	1.562
Glutamine	1.503	0.008	1.573
Glycerol-3-phosphate	0.812	0.008	1.593
Isoleucine	1.209	0.008	1.577
Lactate	0.851	0.008	1.620
Lauric acid	1.371	0.008	1.527
Malic acid	0.273	0.008	1.736
Myo-inositol	2.534	0.008	1.660
Myo-inositol-2-phosphate	1.767	0.008	1.372
Oxalate	2.195	0.008	1.387
Proline	1.763	0.008	1.403
Pyruvic acid	2.655	0.008	1.695
Threonine	1.293	0.008	1.761
UN01	2.983	0.008	1.521
Valine	1.387	0.008	1.722
Citric acid	0.213	0.012	1.653
Ethanedioic acid	2.913	0.012	1.473
Glycine	1.245	0.016	1.363
Heptanoic acid	1.132	0.016	1.316
9-Octadecenoic-acid	2.926	0.032	1.212
Fumaric acid	1.269	0.032	1.382
Glycerol-3TMS	1.180	0.032	1.593
L-Asparagine	1.209	0.032	1.419
Ua8	0.487	0.032	1.188
Uracil	1.266	0.032	1.367

bs, supernatant of the co-culture of anaerobic fungus and methanogen; as, supernatant of the anaerobic fungal monoculture; VIP, variable importance plot.

1) When p<0.05 and VIP>1, there is a significant difference in metabolites between bc and ac.
